# Acute Effect of Percussive Massage on Cross‐Section Area, Muscle Strength, and Late Muscle Pain of the Quadriceps Muscle Following a Fatigue Protocol in Physically Active Men: Randomized Clinical Trial

**DOI:** 10.1002/pri.70246

**Published:** 2026-06-06

**Authors:** Aline da Rosa Castilho, Andreo Fernando Aguiar, Alex Silva Ribeiro, Isadora Fernandes Cônsolo, Fernanda Silvana Pereira Quirino, Rodrigo Franco de Oliveira, Rodrigo Antonio Carvalho Andraus

**Affiliations:** ^1^ Graduate Program in Rehabilitation Sciences Pythagoras Unopar University Londrina Paraná Brazil; ^2^ Faculty of Sports Sciences and Physical Education (FCDEF) University of Coimbra CIPER Coimbra Portugal; ^3^ Graduate Program in Human Movement and Rehabilitation Evangelical University of Goiás (UniEVANGELICA) Anápolis Goiás Brazil

**Keywords:** massage, muscle fatigue, pain, percussion, vibration

## Abstract

**Background and Purpose:**

The massage gun is widely used to alleviate delayed‐onset muscle soreness (DOMS); however, empirical evidence regarding its physiological effects remains limited.

**Objectives:**

This study aimed to evaluate the acute effects of percussive massage on the quadriceps muscle's cross‐sectional area (CSA), strength performance, and pain perception following a fatigue protocol.

**Methods:**

A randomized clinical trial was conducted including 37 physically active men. Participants performed a fatigue protocol on a 45° leg press, at 70% of their one‐repetition maximum (1RM). The volunteers were allocated to an intervention group (G0 *n* = 19, mean ± SD; 25.7 ± 4.5 years, 83.7 ± 10.6 kg, 176.8 ± 6.3 cm) and control group (G1 *n* = 18, mean ± SD; 27.6 ± 5.0 years, 76.8 ± 15.7 kg, 174 ± 7.3 cm). Assessments were performed at baseline, 24 and 48 h, using ultrasound imaging, isometric dynamometer and VAS questionnaire (visual analog pain scale).

**Results:**

Regarding the muscle area, significant increases were observed in the control group *p* < 0,05 for the rectus femoris at both measurement points compared with the baseline, which suggests that percussive massage may mitigate exercise‐induced edema. While no statistically significant differences were observed between groups or across time points for muscle strength, G0 demonstrated a large effect size (*d* > 2.0) in strength maintenance at 24 and 48 h compared to G1. Both groups showed reduction in pain over time, while G0 demonstrated a significant decrease at 48 h compared to 24 h (53%, *p* < 0.001).

**Conclusion:**

Within‐group assessments revealed significant reductions in pain perception over time for the intervention group. Regarding muscle strength, despite a large exploratory effect size observed during recovery, no statistically significant between‐group superiority over passive rest was established. The findings suggest potential preliminary benefits for symptom relief without acute performance deficits.

## 1 Introduction

This study focused on comparing the changes in muscle cross‐sectional area, delayed‐onset pain and quadriceps muscle performance with and without the application of percussive massage in physically active men who underwent strength training sufficient to induce delayed‐onset muscle soreness, which may reduce the performance on daily living activities.

Exercise‐induced muscle fatigue is a consequence of muscle overloading and is a complex physiological phenomenon characterized by a temporary reduction in the neuromuscular system's capacity to generate force or power, frequently followed by Delayed‐Onset Muscle Soreness (DOMS; Beere et al. 2022; Nogueira et al. 2014), commonly affecting exercise practitioners training with higher volumes of repetitions and loads al. Pathophysiologically, DOMS is associated with ultrastructural microlesions in sarcomeres and a subsequent acute inflammatory process. This is clinically manifested with interstitial edema, muscle stiffness, and a nociceptive threshold reduction, ultimately resulting in functional deficits that may persist for up to 7 days (Bonfim et al. 2010; Carvalho et al. 2021). A study conducted by Wang et al. (2021) showed that the muscle cross‐sectional area exhibits an immediate increase followed by muscle fatigue. This phenomenon is attributed to the inflammatory process, which shows a significant reduction at 24 and 48 h after percussive massage stimulation. Delayed‐onset muscle pain (DOMS) starts around 6/8 h after performing physical exercise, reaching its maximum between 24/48 h, lasting up to 5 days (Carvalho et al. 2021), which may cause losses in training routine.

To mitigate these symptoms and accelerate metabolic homeostasis, numerous approaches have been tested, including stretching (Costa et al. 2022), cryotherapies and thermotherapies (Wang et al. 2021), massages (Filippi et al. 2009) and photobiostimulation (Guo et al. 2017). In accordance with a study by (Davis et al. 2021), a systematic review with meta‐analysis, presented that sports massage used for recovery showed improvements in flexibility and DOMS, demonstrating that strength is maintained, potentially improving the perceived readiness; however, it does not result in a true increase in force production, highlighting a significant effect of massages when compared to other stimuli. The proposed mechanism for percussive therapy is based on the application of high‐frequency vibrations that enhance local blood flow and modulate mechanoreceptor excitability, potentially facilitating metabolite clearance and reducing tissue hydrostatic pressure (Mendell 2014; Lu et al. 2019).

The percussive massage is a new modality used worldwide in clinical and sports medicine; however, its effects remain insufficiently understood, and the current scientific literature presents critical gaps. Most investigations are limited to assessing range of motion (ROM), with a lack of robust data regarding the effects of PT on acute muscle morphology—assessed via Cross‐Sectional Area (CSA)—and on the recovery of maximal isometric strength following high‐intensity fatigue protocols. Furthermore, the superiority of PT over passive rest—a condition in which the lymphatic and circulatory systems depend exclusively on intrinsic physiological mechanisms—has yet to be established under rigorous statistical criteria. The main factor attracting interest in this instrument is that it does not require expertise, and the massage gun is simple to operate, consisting of a hand‐held, pistol‐shaped device with an anatomical design. It is non‐invasive, and its applied directly on the sore and/or stiff muscles.

The present study is justified by the need to elucidate whether acute percussive stimuli can modulate inflammatory edema and preserve neuromuscular performance in physically active men. The aim of this study was to compare the effects of Percussive Therapy versus passive rest on the quadriceps muscle cross‐sectional area, peak isometric torque, and perceived pain post‐fatigue. It was hypothesized that acute percussive massage in men would attenuate increases in muscle cross‐sectional area and promote superior recovery of strength and perceived pain at 24 and 48 h compared to the control group.

## 2 Materials and Methods

### 2.1 Sample

**FIGURE 1:**
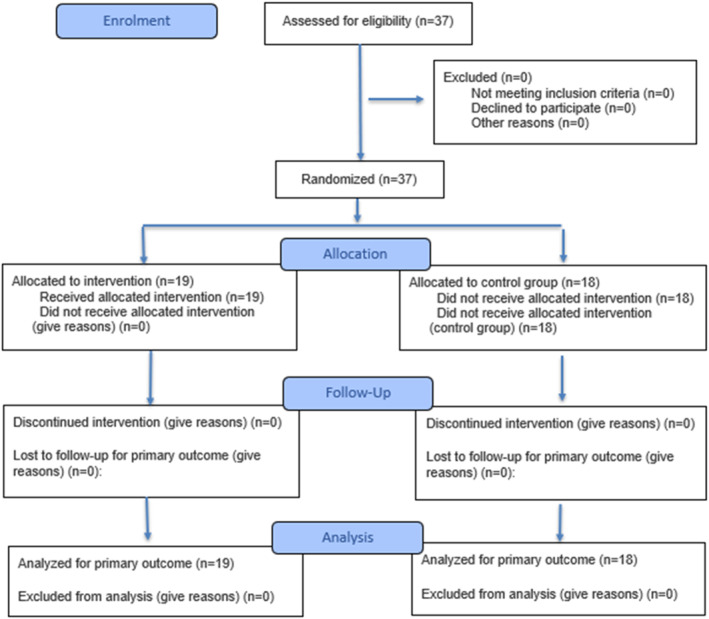
Group allocation diagram (CONSORT).

This is a randomized clinical trial (RCT) with a 1:1 allocation ratio, following the CONSORT guidelines. The study was approved by the Institutional Ethics Committee (Protocol No. 4,889,729) and registered in the Brazilian Registry of Clinical Trials (ReBEC: RBR‐9m5psj7) Held in the city of Londrina, Parana, Brazil. All participants provided written informed consent prior to inclusion. Participants were randomized into the Intervention Group (G0) or Control Group (G1; Figure 1) through a block randomization sequence. Allocation was concealed using sequentially numbered, opaque, sealed envelopes that were opened only after the baseline assessment. Due to the physical and mechanical nature of percussive therapy, true participant blinding during treatment application was not feasible, as the vibratory stimulus was readily apparent. However, participants remained unaware of their specific group sequence assignment during initial baseline collections, and group allocation was completely concealed until the active protocol commenced. The data collection team was not blinded. Thirty‐seven physically active men G0, *n* = 19 (mean ± SD; 25.7 ± 4.5 years, 83.7 ± 10.6 kg, 176.8 ± 6.3 cm); G1, *n* = 18 (mean ± SD; 27.6 ± 5.0 years, 76.8 ± 15.7 kg, 174 ± 7.3 cm), aged 18–40 years were recruited through convenience sampling within the local university community. Participants were included if they performed 150–300 min of physical activity per week according to WHO criteria (Okely et al. 2021). Exclusion criteria included: (a) musculoskeletal injuries in the lower limbs in the last 6 months; (b) use of anti‐inflammatory or analgesic drugs or nutritional supplements that potentially alter muscle recovery (Roveratti et al. 2019); (c) systemic diseases (neurological or metabolic). Convenience sampling was utilized due to the controlled nature of the laboratory setting and accessibility to the target population.

### 2.2 Experimental Draw

#### 2.2.1 Fatigue Protocol

**FIGURE 2:**
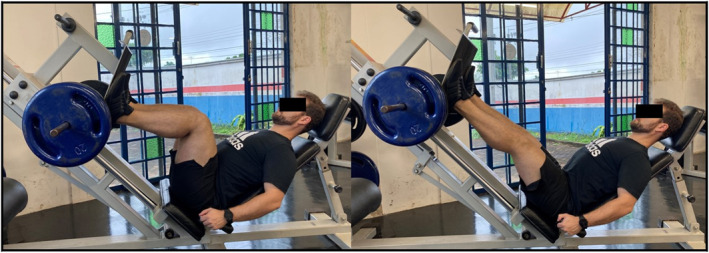
Participants performed the maximum one‐repetition maximum (1RM) on the 45° leg press machine.

The one‐repetition maximum (1RM) was assessed using a 45° leg press machine (Figure 2). The procedure was conducted by three evaluators, two responsible for load control and one for fatigue analysis and time control. It began with a warm‐up of two sets of 15 repetitions at 50% of the participant’s usual load and one set at 100% of the usual training load. After the warm‐up, 10% more weight was added on the leg press, with an additional 5% increase when fatigue reached a rating of 7 on the scale. Participants performed two repetitions with a 3‐min interval between sets, using a range of motion from 90° to 0° of knee extension (Halperin et al. 2015). This process was repeated until the moment the participant was unable to complete the second repetition. At the end of each attempt, the participant was asked about the level of muscular fatigue using the perceived effort scale for resistance exercises (OMNI‐RES) from 0 (zero = extremely easy) to 10 (10 = extremely difficult).

To induce muscle fatigue, 70% of the participant’s 1RM was used (Halperin et al. 2015). Participants performed three sets of failures, with the rest intervals determined by individual recovery needs.

### 2.3 Intervention

#### 2.3.1 Assessment of the Muscle Area—Ultrasonography (USG)

**FIGURE 3:**
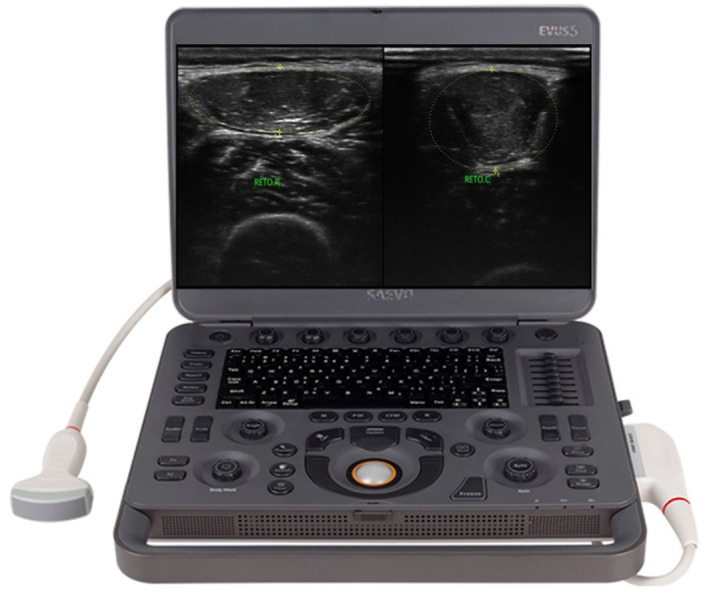
Ultrasound equipment used—SAEVO EVUS5.

To evaluate the image of the muscular area, the SAEVO EVUS5 ultrasound device was used with an L741 transducer with a frequency of 6.4 MHz (Figure 3), conducted by a trained intra‐evaluator. After skin cleansing, the transducer was positioned with a thick layer of conductive gel on the middle third between the origin and insertion of the rectus femoris muscle of the right lower limb (RLL). The images were obtained using the participant in the supine position on the stretcher with the RLL extended and relaxed. The ultrasounds were performed with the muscle relaxed and contracted, respectively, after the evaluator’s command, who captured the image in a transverse section at 2/3 between the iliac crest and the upper edge of the patella. The cross‐sectional area was evaluated by a single reviewer who had received prior training. The rectus femoris was chosen due to its superficial anatomy, which allows easier isolation of muscle boundaries, demonstrating high sensitivity to changes and reliable reproducibility. Since muscle hypertrophy requires 4–12 weeks of training, the changes observed represent the presence of an exercise‐induced inflammatory process (Jae et al. 2015).

#### 2.3.2 Torque Peak—Isometric Dynamometer

**FIGURE 4:**
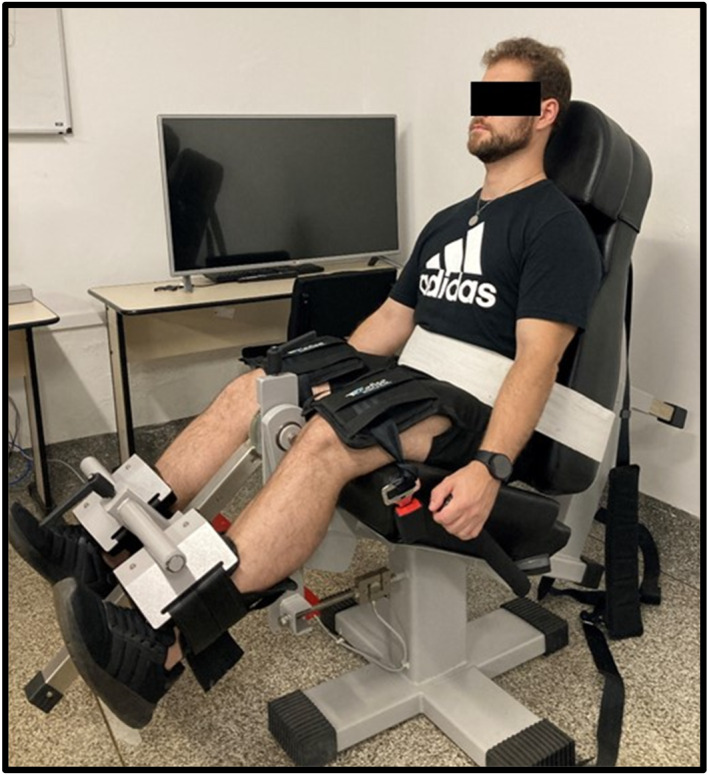
Isometric dynamometer.

To evaluate the maximum voluntary contraction (MVC), a flexo/extensor dynamometer chair developed by cefise biotechnology sports, Ltda (SP) was used (Figure 4), where the data are demonstrated by the N2000 Pro software and transcribed into Excel in kilogram force (kgf) and newtons (N). Participants were positioned seated with their knees at a 30° flexion angle (Davis et al. 2021), where they were instructed to perform 3 maximum knee extensions for 5 s with an interval of 20 s (Krishnan et al. 2010). This angle was chosen following the latest study from 2022 that demonstrate when it is used 30° flexion angle in isometric dynamometer has the same representation if used the dynamometer isokinetic at 60° in flexion angle (Konrad et al. 2020).

#### 2.3.3 Percussive Massage Treatment

**FIGURE 5:**
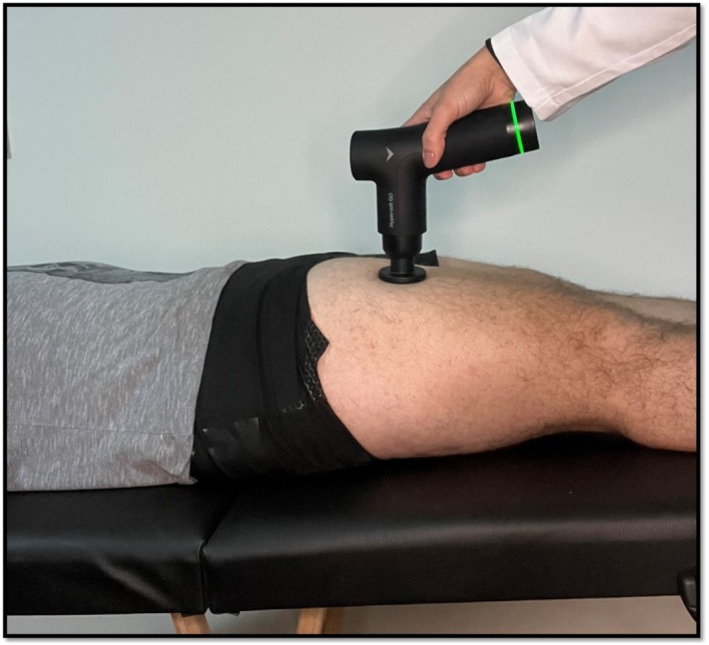
Percussive massage treatment.

With the participant in the supine position on the stretcher, the Hypervolt model GO (Hyperice, n.d.) massage gun was applied by a single previously trained evaluator, who positioned the massage gun over the area, without imposing excessive pressure capable of deforming the tissue, as the deformation occurred only by percussion of the device at a maximum speed of 3200/53 Hz, with a circular and flat tip over the entire quadriceps muscles of both legs, in a vertical direction, going up or down, lasting 5 min on each leg. All participants in the control group maintained the same position for an equivalent duration without receiving the percussive massage. However, at the end of the study, they received the intervention to ensure they did not feel at a disadvantage, in accordance with the study protocol approved by the ethics committee (Figure 5).

#### 2.3.4 Pain Measurement

**FIGURE 6:**
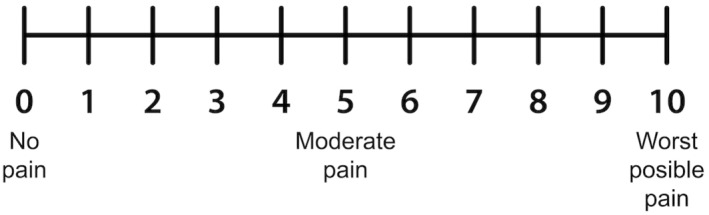
Visual analog scale.

Pain was assessed at 24 and 48 h using the sit‐to‐stand test, as this movement simulates a squat in which hip flexion combined with knee extension produces greater contraction of the rectus femoris, where the individual was positioned seated with hips and knees flexed at 90° and arms crossed in front of the trunk, and was then instructed to stand up and sit down. At the end, he was asked by the evaluator twice without assistance in the hands what score he attributed to his pain on the visual analog scale (VAS), a validated scale sensitive to changes in pain intensity in a subjective manner (Thong et al. 2018), yet capable of converting the individual's experience into a numerical value, allowing for the monitoring of pain progression or reduction before and after interventions (Leabeater et al. 2024; Figure 6).

#### 2.3.5 Statistical Analysis

The sample size was calculate using G*Power software (version 3.0.1; Dusseldorf, Germany) based on a previous study (Sillero et al. 2021) that reported an effect size (ES) of ∼0.6 between manual therapy versus percussion therapy for contractile properties of skeletal muscle during recovery after eccentric exercise. Considering a *α* = 0.05, *β* = 0.80, number of groups = 2, and number of measurements = 3, at least 30 participants (*n* = 15 per group) were required to achieve a statistical power of 82% of rejecting the null hypothesis.

Data normality was assessed using the Shapiro–Wilk test. Descriptive data were presented as mean and standard deviation values or, if they did not meet the normal distribution criteria, as median and interquartile. Comparisons between groups were performed using the student's *t* test or the non‐parametric Mann Whitney test when necessary. To compare the dependent variables between groups and across time points, a two‐way mixed ANOVA (group × time) with repeated measures of the time factor was performed using the general linear model. When a significant effect was identified, post hoc comparisons with Bonferroni correction were applied. To compare the muscle area determined by ultrasound at different moments (baseline, 24 and 48 h), the non‐parametric Friedman test for repeated measures was used, followed by the Bonferroni adjusted multiple comparison test when necessary. The level of statistical significance was set at *p* < 0.05. Effect size was calculated using Cohen’s *d* and interpreted as follows: trivial (< 0.20), small (0.20–0.49), moderate (0.50–0.79), and large (≥ 0.80).

## 3 Results

### 3.1 Cross‐Sectional Area

When comparing the rectus femoris muscular area across time points (baseline, 24 and 48 h), only the control group showed an increase in rectus femoris area, both at rest and during contraction, at 24 and 48 h compared to the baseline measurement (*p* < 0.05), which may be justified by fluid accumulation resulting from the inflammatory process (Table 1).

**TABLE 1:** Intra and intergroup comparisons of the rectus femoris muscle cross‐sectional area.

	Baseline	24 h	48 h	*p* intra	Baseline	*p* inter
Relaxed						0.036
Treated	1.48 (1.19–2.31)	1.59 (1.33–2.29)	1.89 (1.35–3.43)	0.228	24 h	0.001
Control	2.46 (1.50–5.76)	4.87 (3.70–6.28)^a^	5.36 (4.32–6.84)^a^ ^,^ ^b^	0.042	48 h	0.001
Contracted						0.020
Treated	1.52 (1.14–2.47)	1.54 (1.22–2.25)	1.71 (1.25–3.31)	0.504	24 h	0.001
Control	2.49 (1.54–5.38)	4.48 (3.16–7.00)^a^	5.10 (3.77–6.74)^a^ ^,^ ^b^	0.012	48 h	0.001

*Note:* Values are expressed as median and (1st–3rd interquartile range). *p* intra = comparison across time within each group (Friedman test with Bonferroni post hoc), *p* inter = comparison between treated and control groups at each time point (Mann–Whitney test).

^a^ Significant difference compared to baseline within the same group (*p* < 0.05).

^b^ Significant difference compared to 24 h within the same group (*p* < 0.05).

### 3.2 Muscle Strength

There were no significant differences in muscle strength when comparing the groups (intervention and control) or when comparing the moments (baseline, 24 and 48 h). Despite the lack of statistical significance, effect sizes were calculated. The treated group exhibited a very large effect size compared to the control group at both 24 and 48 h, suggesting a clinically relevant preservation of force production (Table 2).

**TABLE 2:** Between‐group comparison of muscle strength.

MS	Baseline	24 h		48 h		ANOVA	*F*	*P*
Groups			*d*		*d*	Time	1992	0.144
Treated	294.6 ± 51.7	292.8 ± 59.6	2.42	302.5 ± 67.0	2.01	Group	1.900	0.177
Control	271.8 ± 61.3	258.9 ± 79.3		273.2 ± 78.3		Interaction	0.430	0.652

*Note:* Variables described as mean and standard deviation.

Abbreviation: MS, Muscle strength in kilograms of force.

### 3.3 Pain Assessment

Both groups reported significant increases in pain levels at 24 h following the fatigue protocol (*p* < 0.001). However, demonstrated a significant effect of time (*F* = 49.94; *p* < 0.001). Although the interaction was not significant, only the treated group showed a significant 53% reduction in pain at 48 h compared to the 24 h assessment (*p* < 0.001), whereas the control group’s pain levels remained statistically similar between 24 and 48 h (Table 3).

**TABLE 3:** Comparison of pain assessed by the VAS during the sit‐to‐stand test.

SRT pain	Baseline		24 h		48 h		ANOVA	*F*	*p*
Groups	Mean	SD	Mean	SD	Mean	SD	Time	49.94	< 0.001
Treated	0	0	2.6^a^	2.2	1.2^a^ ^,^ ^b^	1.4	Group	1.329	0.257
Control	0	0	2.9^a^	2.4	2.3^a^	1.8	Interaction	1.629	0.209

Abbreviation: SRT‐sitting‐rising test.

Significant difference in basal *p* < 0.001.

^a^ Significant difference in basal *p* < 0.05.

^b^ Significant difference in 24 h *p* < 0.001.

## 4 Discussion

The most relevant findings of the present study were: (1) the percussive massage intervention promoted significant stability in the rectus femoris cross‐sectional area in the treated group, whereas the control group exhibited a progressive increase in muscle area at 24 and 48 h. While previous studies (Jae et al. 2015) associate such increases with exercise‐induced muscle damage, we must characterize this phenomenon primarily as extracellular edema rather than a direct measure of inflammation; (2) no statistically significant differences were observed in muscle strength between groups or across time; (3) both groups showed increased pain after the protocol, but the treated group demonstrated a significantly greater reduction in pain at 48 h compared to 24 h, with a 53% reduction, indicating clinically relevant analgesic effects. These findings are discussed below according to each outcome.

### 4.1 Rectus Femoris Cross‐Sectional Area

Regarding muscle morphology assessed by ultrasound, no significant changes were observed in the rectus femoris muscle cross‐sectional area in the treated group over time. In contrast, the control group showed a significant increase at 24 and 48 h compared to baseline, both in relaxed and contracted conditions.

Jae et al. (2015) demonstrated that delayed onset muscle soreness (DOMS) caused by eccentric exercise leads to inflammatory edema and increased muscle thickness; therefore, we must characterize this phenomenon primarily as extracellular edema rather than a direct measure of inflammation. Moreover, Costa et al. (2022), Schoenfeld et al. (2016), and Weiss et al. (2000) reported that structural muscle hypertrophy requires several weeks of consistent training and depends on individual physical and genetic characteristics. Therefore, the acute increase observed in the control group should be interpreted as fluid accumulation rather than structural growth.

The absence of such an increase in the treated group suggests that percussive massage may have contributed to enhanced fluid drainage and modulation of the inflammatory process. However, in the absence of biochemical biomarkers, the claim that percussive massage “controls inflammation” remains speculative and should be interpreted as a modulation of post‐exercise swelling.

### 4.2 Muscle Strength

No statistically significant differences were observed in muscle strength when comparing groups or time points. The hypothesis that percussive massage would enhance or accelerate strength recovery was not supported by the analysis, however, effect size analysis demonstrated very large magnitudes (*d* = 2.42 at 24 h and *d* = 2.01 at 48 h) for strength increase in the intervention group compared to the control group, the discrepancy between *p* values and Cohen's *d* suggests that our sample size (*n* = 37) may have been underpowered to detect inter‐group differences, or that the high standard deviations observed (high inter‐individual variability) masked the effects. Therefore, percussive massage should be viewed as a safe tool that does not impair performance.

Similar findings were reported by Konrad et al. (2020), who applied percussive massage for 5 min at 53 Hz to the calf muscle and found no significant improvements in maximal voluntary contraction. The consistency between studies suggests that short‐duration percussive massage may not acutely enhance strength performance. Corroborating this, Szymczyk et al. (2022) demonstrated that percussive treatment alters local mechanical properties but may not translate into immediate explosive mechanical performance gains.

The discrepancy between statistical significance and large effect sizes in the present study may be related to sample size limitations and high inter‐individual variability. Additionally, as discussed by Filippi et al. (2009), differences in frequency, duration, and application parameters may influence neuromuscular responses to vibratory stimuli. Therefore, further research is needed to clarify the optimal parameters for strength enhancement. Importantly, the intervention did not negatively affect muscle strength, indicating that percussive massage appears safe for use in recovery contexts without impairing performance.

### 4.3 Pain Perception

Pain perception during the sit‐to‐stand test increased at 24 h in both groups, reflecting the expected development of DOMS. However, while both groups showed pain reduction at 48 h, the treated group demonstrated a significantly greater reduction compared to 24 h (*p* < 0.001), corresponding to a 53% decrease.

These findings are consistent with the proposed mechanisms of vibratory stimulation, including increased afferent activity from muscle spindles, modulation of motoneuron excitability, and enhanced local circulation (Mendell 2014; Lu et al. 2019). Lefaucheur (2019) emphasizes that analgesic effects depend on stimulus duration, intensity, location, and device positioning.

While Leabeater et al. (2024) reported no reduction in pain after applying a massage gun to the gastrocnemius for 5 min, the present findings demonstrated significant improvement when applied to the quadriceps. This difference may be related to stimulation frequency rather than duration. Supporting this hypothesis, Lu et al. (2019), in a meta‐analysis of vibratory stimulation studies reported pain improvement at 24 h in 9 out of 10 studies, superiority at 48 h in 8 studies, and continued benefit at 72 h in 6 studies, despite different training protocols used to induce DOMS.

### 4.4 Clinical Implications and Limitations

The present study suggests the clinical applicability of percussive massage for reducing pain perception and modulating inflammatory responses following exercise‐induced muscle damage. The intervention appears to reduce muscle tension without negatively affecting strength performance, making it a potentially useful recovery strategy.

However, important limitations must be considered: the absence of team blinding, the small sample size, the exclusive inclusion of young healthy men, and the lack of comparison between different device types, frequencies, and application durations. These factors limit generalizability and statistical power.

Future studies should include larger and more diverse samples, explore different stimulation parameters, and investigate long‐term adaptations.

### 4.5 Implications of Physiotherapy Practice

The percussive massage may represent a useful adjunct resource in physiotherapy practice for the management of exercise‐induced muscle damage and delayed onset muscle soreness (DOMS). The intervention demonstrated the potential to modulate the inflammatory response, as indicated by the absence of increases in rectus femoris cross‐sectional area in the treated group, which may reflect improved fluid drainage and reduced inflammatory edema. In addition, although no statistically significant differences were observed in muscle strength, the large effect sizes suggest that the technique does not negatively affect strength performance and may even contribute to maintaining neuromuscular function during the recovery period. Importantly, the intervention showed clinically relevant reductions in pain perception at 48 h, indicating that percussive massage may help accelerate symptom resolution and improve patient comfort following intense or unfamiliar physical activity. Therefore, massage guns may be considered a practical, low‐cost, and easily applicable tool that physiotherapists can incorporate into post‐exercise recovery protocols, while recognizing the need to individualize treatment parameters and combine the technique with other evidence‐based rehabilitation strategies.

## 5 Conclusion

The massage gun suggested potential preliminary applicability for modulating pain perception and maintaining muscle cross‐sectional area stability compared with passive rest within this specific sample. Although no statistically significant between‐group superiority was established for muscle strength recovery, percussive massage showed exploratory potential for short‐term symptom management post‐fatigue. However, its role in physiological inflammation and enhancing performance requires further investigation using larger, blinded samples and biochemical markers.

## Funding

The authors have nothing to report.

## Ethics Statement

This study conformed to ethical health research standards and received approval from the Research Ethics Committee (opinion no. 4,889,729), University Pitágoras Unopar Anhanguera—UNOPAR.

## Consent

Informed consent was obtained from all subjects involved in this study.

## Conflicts of Interest

The authors declare no conflicts of interest.

## Data Availability

The entire research protocol is registered in ReBEC (RBR‐9m5psj7).
